# Emergency department transfers and hospital admissions from residential aged care facilities: a controlled pre-post design study

**DOI:** 10.1186/s12877-016-0279-1

**Published:** 2016-05-12

**Authors:** Carolyn Hullick, Jane Conway, Isabel Higgins, Jacqueline Hewitt, Sophie Dilworth, Elizabeth Holliday, John Attia

**Affiliations:** The University of Newcastle, University Drive, Callaghan, NSW 2308 Australia; John Hunter Hospital, Hunter New England Health, Locked Bag 1, HRMC, NSW 2310 Newcastle, Australia; University of New England, Armidale, NSW 2351 Australia; Hunter Medical Research Institute, Locked Bag 1000, New Lambton, NSW 2305 Australia

**Keywords:** Nursing home, Patient transfer, Emergency Department, Integrated care, Model of care, Avoidable admissions, Hospitalization, Acute care, Telephone triage, Clinical handover, Homes for the aged, Pre-post study residential aged care

## Abstract

**Background:**

Older people living in Residential Aged Care Facilities (RACF) are a vulnerable, frail and complex population. They are more likely than people who reside in the community to become acutely unwell, present to the Emergency Department (ED) and require admission to hospital. For many, hospitalisation carries with it risks. Importantly, evidence suggests that some admissions are avoidable. A new collaborative model of care, the Aged Care Emergency Service (ACE), was developed to provide clinical support to nurses in the RACFs, allowing residents to be managed in place and avoid transfer to the ED. This paper examines the effects of the ACE service on RACF residents’ transfer to hospital using a controlled pre-post design.

**Methods:**

Four intervention RACFs were matched with eight control RACFs based on number of total beds, dementia specific beds, and ratio of high to low care beds in Newcastle, Australia, between March and November 2011. The intervention consisted of a clinical care manual to support care along with a nurse led telephone triage line, education, establishing goals of care prior to ED transfer, case management when in the ED, along with the development of collaborative relationships between stakeholders. Outcomes included ED presentations, length of stay, hospital admission and 28-day readmission pre- and post-intervention. Generalised estimating equations were used to estimate mean differences in outcomes between intervention and controls RACFs, pre- and post-intervention means, and their interaction, accounting for repeated measures and adjusting for matching factors.

**Results:**

Residents had a mean age of 86 years. ED presentations ranged between 16 and 211 visits/100 RACF beds/year across all RACFs. There was no overall reduction in ED presentations (OR = 1.17, *p* = 0.56) with the ACE intervention. However, when compared to the controls, the intervention group reduced their ED length of stay by 45 min (*p* = 0.0575), and was 40 % less likely to be admitted to hospital, . The latter was highly significant (*p* = 0.0012).

**Conclusions:**

Transfers to ED and admission to hospital are common for residents of RACFs. This study has demonstrated that a complex multi-strategy intervention led by nursing staff can successfully reduce hospital admissions for older people living in Residential Aged Care Facilities. By defining goals of care prior to transfer to the ED, clinicians have the opportunity to better deliver care that patients require. Integrated care requires accountability from multiple stakeholders.

**Trial registration:**

The Australian New Zealand Clinical Trials Registration number is ACTRN12616000588493 It was registered on 6^th^ May 2016.

## Background

Older people living in Residential Aged Care Facilities (RACFs) are a vulnerable, frail and complex population. The Australian Institute of Health and Welfare reports that 58 % of people living in RACFs in Australia in 2013 were 85 years or older; 82 % have high care needs and 80 % have dementia or mental illness [1].

When compared to older people who do not reside in RACFs, residents in RACFs have a higher proportion of presentations to the Emergency Department (ED), re-presentations to ED, readmission to hospital and increased length of stay (LOS) in both ED and hospital [2]. Reasons for ED transfer include: falls and fall related fractures, cardiovascular and respiratory illness, altered mental state and device related complications such as indwelling catheters. The consequences of transferring older people living in RACFs to ED and admission to hospital is significant with an increased risk of delirium, and other iatrogenic events such as falls, medication errors, pressure injuries, deconditioning and death [3, 4].

When older residents in RACFs need medical care and treatment there is disruption to continuity of care [2, 5, 6] and a need for management of their care across primary, tertiary, community and rehabilitation health services.. When acutely ill, they may be transferred to an ED with little attention to handover [7], including limited documentation of their illnesses; current symptoms or usual presentation. Obtaining a history can be difficult because of cognitive problems. Assessment and evaluation is often complex. Unfortunately many older residents seem to move frequently between acute and long-term care settings. The management of unwell older people within the RACF has been shown to have similar or better outcomes than older people who are managed in hospital [7–11]. When assessing unwell older people, the benefit of hospitalization needs to outweigh the risk [11]. The additional risks associated with hospitalization, without substantial potential benefit for the resident’s clinical course or quality of life necessitate consideration of new or alternative models of care [12, 13]. New approaches to the care of older people are required in order to ensure the care they receive is optimal during periods when they are acutely unwell and not requiring high intensity emergency resuscitation [12, 14]. One approach, which was the focus of this study, was to support enhanced collaborative communication and decision- making between ED and RACF health care teams.

In Australia, care in RACFs is subsidised by the federal government with the majority of services provided by not-for profit organisations. State governments fund acute care services. Registered nurses (RNs) in RACFs account for 15 % of the direct care workforce with the majority of staff being assistants in nursing (AINs) or personal care assistants (PCAs). RNs in RACFs, by virtue of their small numbers provide limited direct care; instead they provide oversight of entire facilities with supervision of AINs and PCAs. Nurse practitioners (NPs) are advanced practice nurses whose numbers are limited and closely controlled by state and federal funding, accounting for only 0.2 % [15] of staff in RACFs despite their apparent success in this setting [16–18]. General Practitioners (GPs) provide medical care, working remotely to the RACF, in their practices and with competing priorities.

The context in which this study occurred was one in which there was no additional funding for RNs in RACFs. In order to develop a new model of care for older people living in RACFs, research was undertaken using focus groups with RACF staff and GPs who visited those facilities to understand the concerns and challenges of managing acutely unwell residents within existing resources. The key points that emerged were that RACF staff, RNs, AINs and PCAs, had a genuine desire to care for their acutely unwell residents. They described the ordeal of transfer for them and the older persons and his or her family, and felt that ED staff did not care for older people adequately, particularly those with dementia. They were concerned about their duty of care to the resident, their lack of expertise in acute illness management and their limited access to medical staff. They asked for guidance and support and recognised that a transfer to the ED might be avoided if they were able to prevent illness deterioration. They voiced a preference for a collaborative approach through telephone support from nursing staff with acute and aged care skills [13]. Based on the findings of Stokoe et al’s study [13] and supported by Arendts and Howard [19] and Crilly et al. [20], a new model of care, the Aged Care Emergency (ACE) service was implemented and evaluated [21].

This paper examines the effects of the ACE service on reducing the RACF residents’ transfer to hospital, admission to hospital and admission length of stay using a controlled pre-post design.

## Methods

### Study design

The study was a controlled pre-post design involving 12 RACFs. Four RACFs with a history of high ED presentations elected to be intervention sites, following their previous participation in a study that identified barriers and facilitators to quality management of acutely unwell residents [13]. For each of the four intervention RACFs, two control RACFs were selected, matched for size (bed number) and RACF type (ratio of high care beds to low care beds and presence of dementia-specific beds). These identifiers were used as they are publically reported in the DPS Guide to Aged Care [22] and represent features that are impacted by RACF policy and staffing, such as provision of Registered Nurses. None of the beds included respite short stay beds or specific specialist beds. The twelve RACFs and their group allocation are shown in Table 1.

**Table 1:** RACF group allocation and matching characteristics

Group	RACF label	Pair	Beds	High to low care bed ratio	Dementia specific
Intervention	Intervention 1	1	108	1.45	No
Control	Control 1a	1	100	1.50	No
Control	Control 1b	1	76	1.92	No
Intervention	Intervention 2	2	72	0.00	Yes
Control	Control 2a	2	84	0.00	Yes
Control	Control 2b	2	53	0.00	Yes
Intervention	Intervention 3	3	142	1.63	Yes
Control	Control 3a	3	120	2.00	Yes
Control	Control 3b	3	124	2.10	Yes
Intervention	Intervention 4	4	131	0.30	Yes
Control	Control 4a	4	153	0.50	Yes
Control	Control 4b	4	126	0.70	Yes

The intervention was provided from March to November 2011 to the four RACFs in the intervention group. Pre-intervention data comprised all ED presentations for the twelve RACFs, both intervention and control, from March 2009 to November 2009 and March 2010 to November 2010. Post-intervention data comprised all ED presentations for the same twelve RACFs from March to November 2011.

### Setting

The setting was a Local Health District in New South Wales, Australia with a range of tertiary and primary services. The tertiary referral hospital, in which the intervention ED is located, is surrounded by a number of RACFs that refer patients to the ED. In the year prior to this study, the ED had 67,000 ED presentations, 13 % of which were by patients over 75 years of age.

Three of the intervention RACFs were not-for-profit organisations and one for-profit, with a total of 413 residents. Three of the intervention facilities had registered nurses onsite 24 h a day. One facility had registered nurses on call overnight. There were no aged care nurse practitioners working in RACFs in the region at the time. The tertiary referral ED was the main ED providing acute care for residents.

### Sample

Outcomes were extracted from electronic hospital admission records based on address fields and verified by reviewing the medical record [23]. This included not only our tertiary care hospital but also the other two local EDs where patients could have been transferred to ensure no ED presentations were missed. Patients were aged 75 years or over at time of ED presentation.

### Intervention

The ACE service model of care comprised

- An ED advanced practice nurse with aged care skills, (ACE Clinical Nurse Consultant) who led and coordinated the ACE service.
- More than twenty evidence based algorithms, developed in consultation with clinical experts, and RACF staff in the region, were standardized for the management of common problems for RACF residents: for example, falls, shortness of breath and indwelling urinary catheter issues. The ACE Clinical Nurse Consultant (CNC) who commenced in October 2010, 5 months prior to the intervention, worked with RACF and ED staff to develop, test and the refine the algorithms and provide education and training. The manual of algorithms guides and supports RACF staff to manage acutely unwell residents in situ and is used as a reference source by RNs, AINs, PCAs, GPs and ED staff [24].
- An education program for clinical staff in RACFs, prior to the introduction of the model supported using the manual of algorithms. The education service provided onsite by the ACE CNC, for the study RACF staff. The program constituted two hours of presentation with ongoing education provide as required or requested.
- An ED RN led telephone consultation service for RACF staff, 12 h during the day, 7 days a week to provide clinical support, assist decision making in the RACF as well as receive clinical handover when the resident required transfer. The four ED RNs were all advanced care nurses with skills and experience in aged care nursing. Their role included identification of care needs, care planning and advocacy for the older person.
- Establishment of the purpose of the ED transfer based on the older person’s goals of care by the RACF staff, with support from the ED Registered Nurse (RN).
- Proactive case management, aligned to the goals of care in the ED by the ED RNs.
- A collaborative respectful relationship with RACFs, ambulance, EDs, GPs and the primary care organisation, working together to achieve optimal patient outcomes.

### Usual care for control RACFs

For acutely unwell residents in the control RACFs, residents are assessed by AINs and PCAs with support from RNs who may not be in the facility. Primary medical care is provided by General Medical Practitioners who visit the RACF as required and have competing demands of a busy practice. There is generally limited handover of clinical information or contact between the RACF and the ED prior to ED arrival. When residents require an ED visit, they are transported by ambulance to and from hospital.

### Outcomes

Efficacy of the intervention was assessed for the five outcomes listed below.

1. ED presentation. For each RACF, the ED presentation outcome was specified as the average number of ED presentations per month during pre- and post-intervention stages. To facilitate statistical modeling, the average monthly count was converted to a proportion with the count as the numerator and the number of beds in the RACF as the denominator. This proportion approximates the probability that a given resident at the RACF will present to ED in a given month. It assumes low turnover of patients within each RACF in any given month.
2. ED length of stay (LOS) in minutes from time of presentation to discharge from ED to either a hospital ward or discharge home to the RACF.
3. Hospital admission following ED presentation
4. Hospital LOS in days was calculated for patients who were admitted to hospital after an ED presentation. It was calculated as the total time spent in hospital from ED presentation to hospital discharge.
5. 28 day hospital re-admission was based on time from one hospital admission to the next. It was calculated for hospitalized patients only.

For each outcome, efficacy was defined as a relative change of the mean or odds of the outcome in intervention RACFs from pre- to post-intervention, compared to the comparable change in control RACFs. ED length of stay, hospital length of stay and 28-day readmission were analysed in order to determine that the intervention did not increase the time patients spent in the ED or hospital and that by avoiding admission, patients were not readmitted more often.

### Study size

Four RACFs were selected for intervention as a practical and feasible number on which to intervene in the time available. Based on the number of ED transfers in the baseline period, we estimated we had 80 % power to detect a 30 % reduction in transfers at a *p*-value of 0.05.

### Statistical analysis

Intervention RACFs and control RACFs were reviewed over the same monthly periods from 2009 to 2011 to eliminate any seasonal variation. Data were analysed by comparing the control and intervention RACFs before and after the intervention, thereby allowing for changes related to time and not the intervention.

Generalized estimating equations (GEE) were used to estimate differential changes between intervention and control RACFs in pre- and post-intervention means (for continuous outcomes) or log-odds (binary outcomes). Differential changes were assessed using a term reflecting the interaction between intervention group (intervention/control) and time (pre/post). This allowed assessment of whether the intervention specifically affected pre-post differences, after accounting for other, unrelated factors that may have also influenced pre-post differences (reflected in control group pre-post differences). Efficacy of the intervention was assessed by testing the two-sided null hypothesis that the interaction term coefficient = 0. The GEE was structured to account for potential correlation between repeated measures (ED presentations) by a single patient, and between patients from the individual RACFs. An exchangeable correlation structure was assumed. Models for outcomes were adjusted for the following covariates to account for the matched nature of the design:

- RACF pair (as a fixed effect)
- RACF bed number
- RACF high care: low care bed ratio

For outcome 1, ED presentation, bed number was incorporated into the outcome variable, so was not indicated as a covariate. Further, RACF pair and high: low care ratio had non-significant (*p* > 0.1) parameter estimates and their removal produced a negligible change in the model. Thus, for parsimony (given fewer observations for this summary outcome measure), no covariates were included in the final model.

Results show estimated parameters, 95 % confidence intervals and *p*-values for the main effects of group (intervention/control) and time (pre/post), and their interaction. Parameters for RACF matching factors are not shown. For continuous outcomes (outcomes 2 and 4), parameters represent the predicted change in the mean outcome for the specified level of group, time, or group × time, compared to the reference group mean. For binary outcomes (outcomes 1, 3, and 5) the parameters are expressed as odds ratios, reflecting the predicted ratio in mean odds of the outcome for specified level of group, time, or group × time, compared to the reference group odds. Associations reaching *p* < 0.05 were considered significant. All statistical analyses were programmed using SAS v9.4 (SAS Institute, Cary, North Carolina, USA).

### Ethical considerations

The study was approved by the Hunter New England Health Human Research Ethics Committee reference no. 11/02/16.5.01; HREC/10/HNE/402; SSA/10/HNE/402 in February 2011. According to the ethics committee, consent from individual patients was not required since:

- data were analysed at the cluster level (RACF)
- consent to participate was obtained at the cluster level
- individuals within each cluster were de-identified
- data were routinely collected through hospital administrative data, without the need to approach individuals.

### Trial registration

The Australian New Zealand Clinical Trials Registration number is ACTRN12616000588493 It was registered on 6^th^ May 2016.

## Results

The mean age of patients was approximately 86 years in both treatment groups, pre- and post-intervention, as described in Table 2. 19.8 % of control patients and 19.9 % of intervention ED presentations were related to falls. The next most common presenting problem was shortness of breath and respiratory illness, with 11.3 % in the control group and 11.6 % in the intervention group. This is further outlined in Table 2

**Table 2:** Patient and RACF characteristics pre- and post-intervention within intervention groups. Pre-period reflects 2 years, where as post period reflects 1 year

Characteristic	Time	Control	Intervention
Age: mean (SD) Years	Pre	86.1 (5.4)	85.9 (5.2)
	Post	85.9 (5.3)	86.0 (5.1)
Annual ED presentations: N	Pre	653	604
	Post	468	525
Mean ED length of stay (Minutes) (SD)	Pre	496.7 (302.7)	496.3 (267.3)
	Post	481.7 (331.1)	435.7 (315.9)
Annual Hospital admissions: N	Pre	399.5	399
	Post	317	312
Mean Hospital length of stay (Days): mean (SD)	Pre	10.0 (14.8)	9.4 (13.7)
	Post	8.0 (11.0)	6.3 (8.5)
Annual Individual patients: N	Pre	415	360
	Post	246	172
Presentations per patient: mean (SD)	Pre	1.55 (1.0)	1.66 (1.1)
	Post	1.87 (1.4)	2.46 (1.6)
28 day hospital re-admission: N	Pre	74.5	82.5
	Post	55	84
Presenting problem (%)	Fall	19.8 %	19.9 %
	Respiratory	11.3 %	11.6 %
	Abdominal	8.6 %	11.6 %
	General	11 %	6.9 %
	Cardiac	8 %	8.8 %
	Pain	8 %	6.5 %
	Other	33.3 %	34.6 %

### ED presentations

Table 2 describes the annual ED presentations for both the intervention and control RACFs. Table 3 illustrates the mean monthly ED presentations by individual intervention RACFs in comparison to their control RACFs. The intervention RACFs show higher monthly presentation values, consistent with their selection as the initial sites that might benefit the most from such an intervention. When analysing both the impact of time and the matched controls, the non-significant parameter estimate for the Group × Time interaction suggests that patients from intervention RACFs and control RACFs had a similar change in the odds of ED presentation in any given month pre- to post-intervention (OR = 1.17, *p* = 0.56). This suggests lack of efficacy of the intervention for achieving a relative reduction in the odds of ED presentation, compared to control RACFs.

**Table 3:** Mean monthly ED presentations pre- and post-intervention for each RACF

Group	RACF label	Pair	Beds	Mean monthly presentations pre	Mean monthly presentations post	Presentations/100 RACF beds/year post
Intervention	Intervention 1	1	108	13	8	93
Control	Control 1a	1	100	16	11	132
Control	Control 1b	1	76	6	4	63
Intervention	Intervention 2	2	72	11	8	142
Control	Control 2a	2	84	7	5	65
Control	Control 2b	2	53	9	7	169
Intervention	Intervention 3	3	142	23	25	211
Control	Control 3a	3	120	14	11	107
Control	Control 3b	3	124	2	3	16
Intervention	Intervention 4	4	131	20	16	150
Control	Control 4a	4	153	13	7	58
Control	Control 4b	4	126	6	5	50

### ED length of stay

**Fig. 1:**
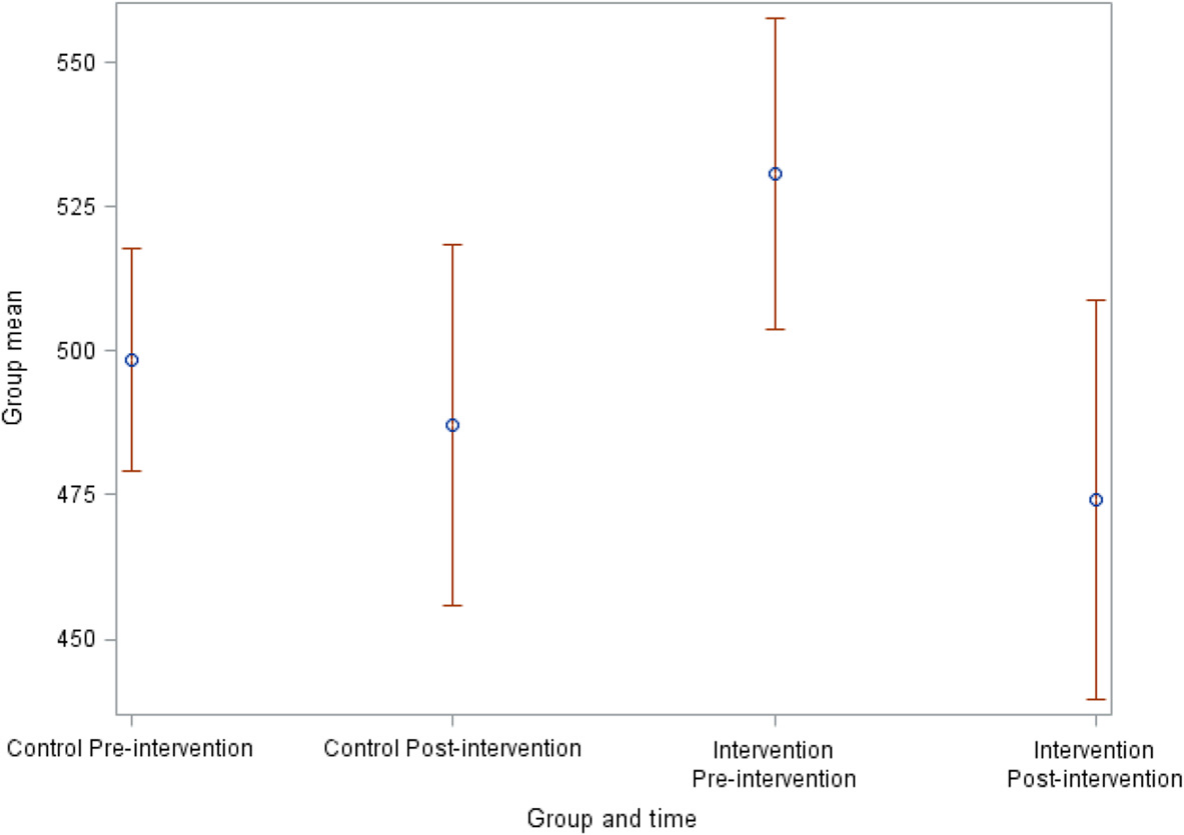
ED Length of stay tended to improve more in the intervention group with an additional 45-min reduction

Table 2 describes the change in mean ED length of stay for both intervention and control RACFs with control RACFs ED length of stay reducing from 496.7 min to 481.7 min while the intervention RACFs ED length of stay reduced further from 496.3 min to 435.7 min. Table 4 shows that when the data was adjusted for matching factors, the intervention group had a longer estimated ED length of stay pre intervention, by 32 min on average. Over time, both groups tended to stay in the ED less time. The mean reduction was 11 min in controls. However, the interaction term (Group X Time) indicates that the intervention RACFs reduced their ED LOS over time more than the control RACFs, decreasing by an extra 45 min. This was of borderline statistical significance (*p* = 0.0575), showing marginal efficacy. Figure 1 shows this graphically, indicating that although intervention RACFs started with a higher ED LOS than control RACFs, they ended up with a lower ED LOS post-intervention.

**Table 4:** Results for reduction in ED length of stay (minutes)

Parameter	Value	Estimate	95 % Lower Confidence Limit	95 % Upper Confidence Limit	*p*-value
Group (ref = Control)	Intervention	32.2706	−0.3321	64.8732	0.0524
Time (ref = Pre-intervention)	Post-intervention	−11.2240	−46.2773	23.8292	0.5303
Group x Time interaction	Intervention group, post-intervention	−45.4602	−92.3731	1.4527	0.0575

### Hospital admission

Table 2 describes the annual hospital admissions for both intervention and control RACFs. Table 5 shows that across both pre- and post-intervention periods, intervention RACF patients had about 59 % greater odds of hospital admission than control patients, with this difference being highly significant (*p* = 0.0002). The odds of hospital admission tended to increase (by ~35 %) from pre- to post-intervention across all RACFs, with this increase being significant (*p* = 0.01).

**Table 5:** Results of hospital admission following ED presentation

Parameter	Value	Odds ratio	95 % lower limit	95 % upper limit	*p-*value
Group (ref = Control)	Intervention	1.588	1.247	2.022	0.0002
Time (ref = Pre-intervention)	Post-intervention	1.346	1.072	1.691	0.0106
Group x Time interaction	Intervention group, post-intervention	0.589	0.427	0.812	0.0012

Group × Time interaction suggests that although both groups tended to increase in the odds of hospital admission, the increase was significantly less in intervention RACFs (by ~40 %), with this relative difference being highly significant (*p* = 0.0012). This suggests efficacy of the intervention for achieving a reduction in the odds of hospital admission, compared to control RACFs.

### Hospital length of stay

The hospital length of stay tended to decrease post-intervention in both treatment groups, but to a greater extent in intervention RACFs with their length of stay reducing from 9.4 days to 6.3 days after the intervention, as described in table 2, compared to the control RACFs that reduced from 10.0 days to 8.0 days. The parameter estimate for the Group × Time interaction suggests that although both groups tended to decrease in LOS, the decrease was greater in intervention RACFs (by 1.36 days), although the relative difference was non-significant (*p* = 0.18).

### 28-day hospital readmission

Table 2 describes the numbers of patients who were readmitted within 28 days of hospital admission. Table 6 shows the parameter estimate for the Group × Time interaction suggesting that patients from intervention RACFs and control RACFs had a similarly negligible change in the odds of 28 day hospital admission pre- to post-intervention (OR = 1.18, *p* = 0.49). 28-day hospital re-admission decreased in both groups, but to a lesser extent in intervention RACFs.

**Table 6:** Results for 28-day hospital re-admission

Parameter	Value	Odds ratio	95 % lower limit	95 % upper limit	*p*-value
Group (ref = Control)	Intervention	0.937	0.665	1.321	0.7113
Time (ref = Pre-intervention)	Post-intervention	1.064	0.738	1.533	0.7410
Group x Time interaction	Intervention group, post-intervention	1.177	0.737	1.878	0.4948

## Discussion

The aim of the intervention was to reduce the number of transfers and admissions for acutely unwell residents from the RACF transferred to the ED through the ACE service. The patient data demonstrated an average age of 86 years, consistent with the Australian national data [1]. Most of the study RACFs regularly transferred residents to hospital. This range showed large variation, ranging from 16 to 211 transfers per 100 RACF beds per year. With only one RACF transferring less than 50 patients per 100 RACF beds per year, this is much higher than the national data that suggests 1 in 4 RACF residents have at least one hospital admission per year [1], assuming that RACF residents have a low turnover during the year. The systematic review by Arendts reported rates from seven countries at 10 to 150 transfers per 100 RACF beds per year [23] suggesting rates of more than 30 transfers per 100 beds per year. None of the Australian studies reviewed reported the data in this manner. In our study, RACF transfer rates tended to be higher than previously reported, suggesting an expectation of continued improvement under the ACE service.

As noted previously, the four intervention RACFs were selected because they had been identified as having high rates of hospital transfer and admission [13]. Prior to the introduction the ACE service, the intervention RACF patients were 59 % more likely to be admitted to hospital, compared to the control RACFs. Although all RACFs tended to increase their admission rate over time, the intervention mitigated this, with results indicating that this increase was 40 % less than the control group (*p* = 0.0012). By defining the purpose of the hospital transfer and the patient’s goals of care prior to sending the patient to the ED and active case management in the ED, admission could be avoided. High-quality medical decision-making requires weighing the risks and benefits of treatment options against the primary goal of care [11].

Clinical Handover allows ED staff to prioritise the care that is required by the RACF clinical team. Without this information, EDs are confronted with complex, frail patients who they have limited information about. The patients are often transferred alone with no one to advocate for their care. In line with the ACE service model of care, by developing relationships and opening communication between the intervention RACFs and ED, there was a clearer understanding of the purpose of the transfer and what needed to be done by the ED. Furthermore, the opportunity to send patients home, rather than admit them to hospitals, in line with the patient and their families’ wishes was facilitated. For example, if a patient had a fall, it could be agreed that the resident is transferred to the ED for imaging of their hip but not management of their longstanding, complex medical problems which are best managed by the GP.

The number of ED presentations did not significantly change, (*p* = 0.56). Most facilities, whether intervention or control, reduced ED presentation over the intervention period, making the impact of the intervention less clear. By focussing on care for residents for the four intervention facilities, there may have been heightened awareness for all RACF patients, influencing the ED clinicians’ decision-making. The impact on hospital admission rather than ED presentation was consistent with other research where hospital admission was more likely to improve than ED transfer rates [23, 25]. Prior to the intervention, the patients stayed on average over 8 h in the ED, similar to prior research [3]. This was reduced by 45 min more in the intervention group than the control group, despite more attention in the ED. Although ED LOS was marginally significant, (*p* = 0.0575), with significant pressure on EDs from overcrowding, 45 min reduction in length of stay could be considered clinically important. This was all the more remarkable given that the underlying trend was for greater admission to hospital over time. Similarly, hospital length of stay was reduced for the intervention group, though this was not significant (*p* = 0.18). Again, a reduction from 9.4 days to 6.3 days seems clinically important.

Previous research includes those focused on single strategies: ensuring appropriate clinical management within the facility supported by relevant clinical guidelines, particularly pneumonia [23]; effective and accessible primary care provision within the facility; standardizing communication and clinical handover protocols [26, 27]; enhanced use of advance care directives; and effective care planning and preventative care [10, 23, 27].

INTERACT and OPTIMISTIC are two programs, based in the US, that focus on improving the identification, evaluation, and management of acute changes in condition of nursing home residents. Tools include communication as well as clinical algorithms for common nursing home conditions. They describe leadership, engagement of direct care staff and a culture of quality improvement as keys to success [25, 28]. These elements have commonality with the ACE service though the ACE intervention focussed on acutely unwell residents and clearer relationships with the acute hospital ED, not prevention. This acute service was available to provide support RACFs when the older person’s care needs were urgent and the facility was considering transfer to hospital. The ACE CNC coordinates the service along with regular interagency meetings involving ED, ambulance and RACF staff as well as education and training in the clinical guidelines. No studies describe the use of nurse led telephone support for RACFs.

The ACE intervention is novel. The accountability of care for acutely unwell RACF residents lies with multiple stakeholders. The strength of the ACE service is in empowering RACF staff to manage the residents themselves with support from acute hospitals as well as general practice and ambulance service. Given the complexity of care needs and the challenges of assessments as well as the need to understand the individual goals of care for residents and their families, the model of care brings together many stakeholders to address residents’ needs.

### Limitations

Identification of patients living in RACFs is challenging in hospital administrative data as patient address is used. By not being able to identify patients, hospital planning and management for RACF patients and their distinct clinical needs is difficult. As a result of this study, a unique identifier for RACFs has been added to our local health record. This may also explain some of the discrepancy with national data [1] and the literature [23] as it is possible the issue of identification in hospital administrative data is larger than our local region. The selection of the intervention RACFs on the basis of frequent previous ED transfers could be seen as a form of selection bias, however the direction of this bias would be towards the null, i.e. reduce the likelihood of seeing an effect of the ACE program. Furthermore, to ensure that the results were not simply regression to the mean, the pre-post comparison in these intervention RACFs was also compared to control RACFs and pre values based on 2 years’ worth of data.

### Practice/future

The ACE service has continued to evolve from this pilot to now cover eight EDs and 120 RACFs. The local Primary Health Network has accountability for supporting integrated care. Without a shared accountability between acute care hospitals, RACFs and older people and their families, management of acutely unwell residents can be less than ideal. RACFs can be led to believe that the responsibility lies with ambulance and ED and ED believe the responsibility lies with RACFs and GPs.

The Primary Health Network has supported further development of the ACE service to provide telephone support 24 h a day, seven days a week as well as nurse educators to support the RACFs. [29] Future work includes providing performance reports to RACFs and EDs in order to better define service needs as well as a health economic evaluation.

## Conclusions

Transfers to ED and admission to hospital are common for residents of RACFs. This study has demonstrated that a complex multi-strategy intervention coordinated by nursing staff can successfully reduce hospital admissions for older people living in Residential Aged Care Facilities. By defining goals of care prior to transfer to the ED, clinicians have the opportunity to better deliver care that patients require. Integrated care requires accountability from multiple stakeholders.

### Ethics approval and consent to participate

The study was approved by the Hunter New England Health Human Research Ethics Committee reference no. 11/02/16.5.01; HREC/10/HNE/402; SSA/10/HNE/402 in February 2011. According to the ethics committee, consent from individual patients was not required since:

- data were analysed at the cluster level (RACF)
- consent to participate was obtained at the cluster level
- individuals within each cluster were de-identified
- data were routinely collected through hospital administrative data, without the need to approach individuals.

### Consent for publication

Not applicable.

### Availability of data and materials

De-identified hospital administrative data is available from the lead author upon request.

## Abbreviations

ACE: Aged Care Emergency
ACE: CNC Aged Care Emergency Clinical Nurse Consultant
AIN: assistant in nursing
ED: Emergency Department
GEE: generalized estimating equations
GP: general practitioner
INTERACT: interventions to reduce acute care transfers [25]
LOS: length of stay
NP: nurse practitioner
OPTIMISTIC: Optimizing patient transfers, impacting medical quality, and improving symptoms, Transforming intuitional care project [28]
PCA: personal care assistant
RACF: Residential Aged Care Facility
RN: registered nurse
